# C-855-04. Early Primary Care Follow-Up and Long-Term Mortality in Burn Survivors: A Population-Based Cohort Study

**DOI:** 10.1093/jbcr/irag033.088

**Published:** 2026-04-06

**Authors:** Darby Little, Elliott K Yee, Barbara Haas, Laura Rosella, Gemma Postill, Liisa Jaakkimainen, Stephanie A Mason

**Affiliations:** University of Toronto, Toronto, ON; University of Toronto, Toronto, ON; University of Toronto, Toronto, ON; University of Toronto, Toronto, ON; University of Toronto, Toronto, ON; University of Toronto, Toronto, ON; University of Toronto, Toronto, ON

## Abstract

**Introduction:**

Major burn injury survivors have complex long-term sequelae and are at high risk of long-term mortality. In other hospitalized populations, early follow-up with a primary care provider (PCP) has been associated with improved long-term survival. We hypothesized that early PCP follow-up post-discharge is associated with improved post-discharge survival in burn survivors.

**Methods:**

We performed a retrospective, population-based cohort study of adults attached to a PCP who survived hospitalization for a major burn injury between 2010 and 2022. The exposure of interest was a visit with a patient’s own PCP within 30 days of discharge. The primary outcome was one-year all-cause mortality. Cox proportional hazards models were used to estimate the association between early PCP follow-up and one-year mortality, adjusting for age, sex, comorbidities, rurality, and socioeconomic characteristics.

**Results:**

Among 1690 burn survivors (71% male, median [IQR] age 48 [27] years) attached to a PCP prior to injury, 40% of patients had an early PCP follow-up visit. Those with early PCP follow-up were older, more often female, living in rural areas, and had greater comorbidity burden. One-year mortality was 4% overall; 3% for those with early PCP follow-up, and 5% for those without (*p*=.02). After covariate adjustment, early PCP follow-up was associated with a 65% reduction in one-year mortality (HR = 0.35, 95% CI 0.20–0.62). Early PCP follow-up was more protective in older adults (≥65 years) and larger burns (≥10% TBSA).

**Conclusions:**

Early primary care follow-up was associated with greater one-year survival in burn survivors, particularly in older adults and in those with larger burns.

**Applicability of Research to Practice:**

Burn centres should prioritize the coordination of primary care follow-up as a strategy to support burn survivors post-discharge.

**Funding for the study:**

Innovation Fund Grant.

